# In vivo biomolecular imaging of zebrafish embryos using confocal Raman spectroscopy

**DOI:** 10.1038/s41467-020-19827-1

**Published:** 2020-12-02

**Authors:** Håkon Høgset, Conor C. Horgan, James P. K. Armstrong, Mads S. Bergholt, Vincenzo Torraca, Qu Chen, Timothy J. Keane, Laurence Bugeon, Margaret J. Dallman, Serge Mostowy, Molly M. Stevens

**Affiliations:** 1 Department of Materials, Department of Bioengineering and Institute of Biomedical Engineering, Imperial College London, London, SW7 2AZ UK; 2 Department of Infection Biology, London School of Hygiene & Tropical Medicine, Keppel Street, London, WC1E 7HT UK; 3 Department of Life Sciences, Imperial College London, London, SW7 2AZ UK; 4 Present Address: Department of Craniofacial Development & Stem Cell Biology, Kings College London, Tower Wing, Guy’s Hospital, London, SE1 9RT UK

**Keywords:** Zebrafish, Imaging, Raman spectroscopy

## Abstract

Zebrafish embryos provide a unique opportunity to visualize complex biological processes, yet conventional imaging modalities are unable to access intricate biomolecular information without compromising the integrity of the embryos. Here, we report the use of confocal Raman spectroscopic imaging for the visualization and multivariate analysis of biomolecular information extracted from unlabeled zebrafish embryos. We outline broad applications of this method in: (i) visualizing the biomolecular distribution of whole embryos in three dimensions, (ii) resolving anatomical features at subcellular spatial resolution, (iii) biomolecular profiling and discrimination of wild type and ΔRD1 mutant *Mycobacterium marinum* strains in a zebrafish embryo model of tuberculosis and (iv) in vivo temporal monitoring of the wound response in living zebrafish embryos. Overall, this study demonstrates the application of confocal Raman spectroscopic imaging for the comparative bimolecular analysis of fully intact and living zebrafish embryos.

## Abstract (web summary)

Raman spectroscopic imaging (RSI) can provide information on the chemical composition of a sample, but application to living organisms has lacked sufficient spatial resolution and signal strength. Here the authors apply confocal RSI to whole-mount zebrafish embryos to distinguish different infectious bacteria and to living zebrafish embryos to monitor the wound healing process.

## Introduction

Zebrafish (*Danio rerio*) embryos are an important model organism with a rapid, external development and high optical transparency that enables direct, visual access to biological processes. The majority of zebrafish research is conducted using fluorescence imaging techniques, such as confocal fluorescence microscopy^1^, multiphoton excitation microscopy^2^, or light-sheet fluorescence microscopy^3,4^. These imaging approaches usually involve the detection of artificial fluorescent labels (e.g., transgenic proteins, antibodies) rather than visualizing intrinsic properties of the embryo. While this allows certain biomolecules to be probed with high specificity, sample labeling provides an incomplete picture of the specimen biochemistry. The inability to simultaneously visualize complex biomolecular features represents a major limitation of fluorescence imaging. For instance, zebrafish embryos are commonly used to study bacterial infections and cancers^5–7^; however, single labels cannot be used to access the complex biomolecular variations that determine how different disease phenotypes respond to treatment^8–12^. Moreover, while label-free imaging techniques such as second and third harmonic generation microscopy have been successfully used to image structural features of zebrafish^13,14^, they are unable to provide specific biomolecular information. It is evident that there is an unmet need for a high-resolution imaging technique capable of visualizing and analyzing local biomolecular heterogeneity in different zebrafish embryo models.

Raman spectroscopic imaging is a microscopy technique that utilizes inelastic scattering of laser light to generate spectroscopic datasets, which can then be used to directly map chemical bonds in unlabeled samples^15^. This method can be used to locally identify a broad range of biomolecules using a single excitation wavelength laser, and when combined with multivariate component analysis, it can be used to resolve complex biomolecular features with subcellular spatial resolution^16^. Raman spectroscopic imaging has previously been used to create two-dimensional (2D) and three-dimensional (3D) biomolecular maps of individual cells, enabling highly accurate classification on the basis of biological origin, strain, phenotype, metabolic state, or topography^16–18^. Despite these apparent benefits, there are only a limited number of examples in which Raman spectroscopy has been applied to zebrafish embryos. A few studies have reported the collection of individual point spectra to analyze bone maturation in zebrafish embryos^19,20^, but while this method is rapid, it does not provide any spatial information. Nonlinear Raman methods have been used for imaging^21,22^, however, the standard implementation of these approaches amplifies specific signals and does not provide full spectral coverage. Two Raman techniques are particularly suited for spatially resolved hyperspectral imaging of zebrafish embryos: those employing either light sheet or confocal-based imaging. Oshima et al. demonstrated the use of light sheet-excited spontaneous Raman spectroscopy (LSDRS) to image live medaka fish (*Oryzias latipes*) embryos, a related fish model. However, this technique was limited by a low spatial resolution and weak signal^23^. More recently, Muller et al. used light sheet Raman micro-spectroscopy (LSRM) with significantly improved resolution and signal-to-noise ratio to image the eye of a fixed zebrafish embryo^24^. Furthermore, light-sheet Raman spectroscopy has been used to image cryosections of zebrafish embryo eyes^25^.

The work presented here demonstrates how high-resolution confocal Raman spectroscopic imaging (cRSI) can be employed for analysis of whole-mount zebrafish embryos. cRSI offers an unsurpassed combination of z-resolution and signal-to-noise ratio^24^, features that are highly advantageous for the biomolecular analysis of zebrafish embryos. Here, we present an extensive methodology that enables unique access to the full complement of biomolecular information in both living and fixed whole-mount zebrafish embryos. We generate 3D images of whole zebrafish embryos as well as high-resolution anatomical images revealing fine biomolecular details, including single-cell nuclei and individual muscle fibers. Importantly, the collection of Raman spectra allows us to obtain spatially resolved, quantitative information from a broad range of biomolecules (e.g., lipids, proteins, nucleic acids). We employ multivariate component analyses to extract biomolecular profiles for the interrogation of local biomolecular heterogeneities. For instance, we show that volumetric cRSI can distinguish between lesions formed from two different *Mycobacterium marinum* strains in a zebrafish embryo model of tuberculosis. We also demonstrate that in vivo time-lapse cRSI can be used to temporally monitor the wound response in living zebrafish embryos. Overall, this study shows how Raman-based imaging enables comparative biomolecular analysis using zebrafish embryo models.

## Results

### Development of cRSI of zebrafish embryos

**Fig. 1:**
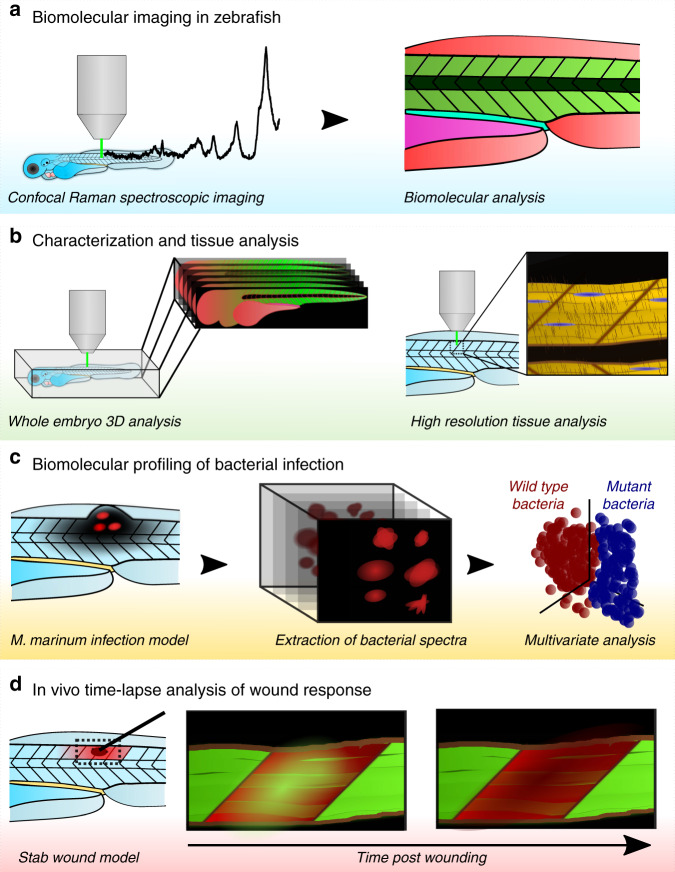
Schematic overview of the confocal Raman spectroscopic imaging applications in zebrafish embryos developed in this study. **a** Confocal Raman spectroscopic imaging (cRSI) is a label-free imaging technique that uses molecular vibrations from Raman scattering to generate biomolecular images of the zebrafish embryo. **b** Characterization and tissue analysis of zebrafish embryos. cRSI can provide three-dimensional biomolecular information from an entire zebrafish embryo or high-resolution imaging of specific tissue regions. **c** Biomolecular profiling of bacterial infection. cRSI can be used to probe local biomolecular variation in bacterial populations after infection. We demonstrated this approach by using volumetric cRSI to discriminate between wild type and region of difference 1 (ΔRD1) mutant *Mycobacterium marinum* in a zebrafish infection model. **d** In vivo time-lapse analysis of wound response. In vivo cRSI can be used to collect Raman spectroscopic images at multiple time points in living embryos and used for time-lapse biomolecular analysis. We demonstrated this concept by following a wound response model for 12 h in living zebrafish embryos.

We developed and optimized a comprehensive workflow of sample preparation, data acquisition and multivariate component analyses for zebrafish research (Fig. 1). We used *TraNac* zebrafish embryos (*mitfa*^*w2/w2*^, *mpv*^*b18/b18*^)^26^, an optically transparent mutant line lacking iridophore and melanophore pigments, which were oriented on a glass slide and mounted in low melting point agarose. cRSI was then performed using a ×63 objective lens and a 532 nm laser (see “Methods” for more details). This sample preparation and confocal imaging setup enabled the acquisition of high-quality Raman spectra with minimal interference from either endogenous pigments or out-of-plane background signal. From these spectra we identified several peaks that have previously been assigned to biomolecules, such as lipids, protein, and nucleic acids. We have provided a full library of these Raman peaks, with details of the tentatively assigned molecular vibration, the associated biomolecular compound and the observed anatomical location in the zebrafish embryo (Supplementary Table 1)^27–37^. This annotated peak library was used throughout this work for biomolecular analysis of the cRSI datasets.

### Volumetric cRSI biomolecular imaging

**Fig. 2:**
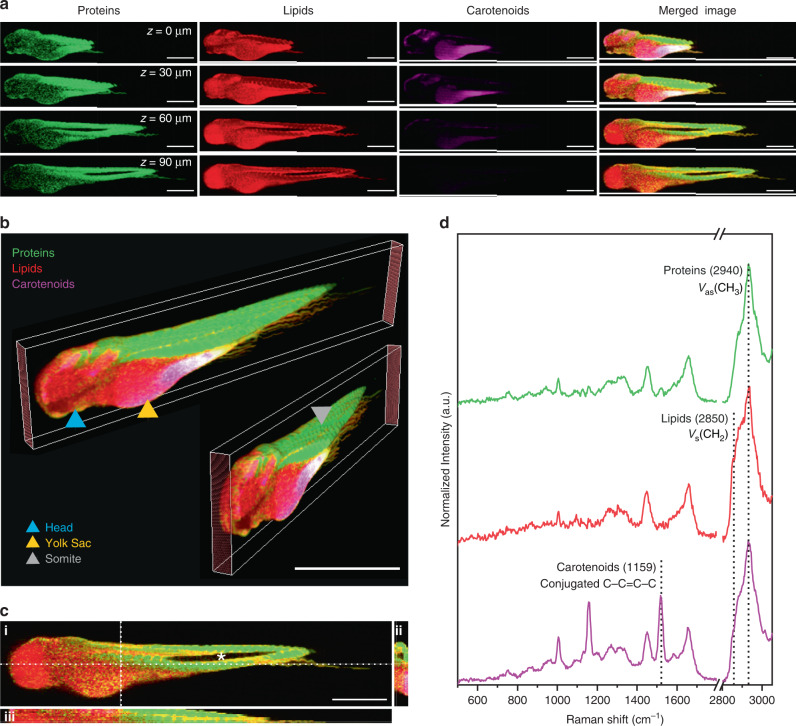
Volumetric confocal Raman spectroscopic imaging of whole zebrafish embryos. Confocal Raman spectroscopic imaging (cRSI) was used to image a fixed embryo (*N* = 1) at 3 days post fertilization at 10 μm in-plane resolution and 10 μm out-of-plane resolution. **a** Single images collected at different confocal planes with normalized univariate peak intensity (a full dataset with all confocal planes is shown in Supplementary Fig. 1). The Z-position relative to the top slice of the stack is indicated. Scale bars: 500 μm. **b** These individual confocal planes were reconstructed into a multichannel 3D stack with 10 x 10 x 10 µm^3^ voxel resolution, enabling whole-embryo visualization at different angles. The blue triangle indicates the head, the orange triangle indicates the yolk sac, and the grey triangle indicates a somite. Scale bar: 1 mm. **c** *Z*-projections were also generated enabling cross-sectional visualization of (i) the sagittal plane, (ii) the frontal plane and (iii) the transverse plane. The asterisk in the sagittal plane denotes the hollow notochord, which can also be visualized as a cross-section in the transverse plane images. Scale bar: 500 µm. **d** Representative spectra collected from volumetric cRSI performed on whole zebrafish embryos. The annotated peak centers used for univariate analysis are indicated by dotted lines. Univariate analysis was performed by integrating over a wavenumber range corresponding to relevant biomolecules: protein-rich regions at 2940 ± 16 cm^−1^ (shown in green), lipid-rich regions at 2850 ± 5 cm^−1^ (shown in red), carotenoid-rich regions at 1159 ± 16 cm^−1^ (shown in magenta).

We demonstrated this workflow by performing a whole-body volumetric scan of a zebrafish embryo fixed 3 days post-fertilization (Fig. 2a and Supplementary Fig. 1). The yolk sac, vasculature, and outer surface of the embryo exhibited a degree of autofluorescence, while a weaker Raman signal was observed in the head region. However, these imaging artifacts were minimal and localized, which enabled us to extract well-resolved biomolecular information from the rest of the embryo. 3D stacks (10 µm lateral and 10 µm axial resolution) were reconstructed using univariate analysis of Raman peaks corresponding to key biomolecular components, which allowed the whole zebrafish embryo to be observed through the sagittal, transverse, and frontal planes (Fig. 2b–d). For the univariate analysis, we used a strong Raman peak centered at 2940 cm^−1^ (*ν*_as_ CH_2_) corresponding to the presence of protein. This signal was visible throughout the embryo and was particularly useful for demarcating the individual body segments, known as somites. We also detected a strong lipid signal centered at 2850 cm^−1^ (*ν*_as_ CH_2_), clearly visible at the junction between somites, which was also the predominant biomolecular feature of the yolk sac, the front of the head, and the neural tube. In addition, we observed strong pigment peaks around the outer layers of the embryo centered at 1159 and 1528 cm^−1^ (conjugated C–C=C–C stretch). Although *TraNac* embryos are deficient in iridophores and melanophores^26^, they do produce a third class of pigments, known as xanthophores. Moreover, zebrafish embryos contain maternally derived β-carotene in the yolk sac, which is used in the biosynthesis of retinoic acid^38^. It is likely that the sharp pigment peaks observed in the cRSI arise from these carotenoid molecules (xanthophores, β-carotene, retinoic acid).

**Fig. 3:**
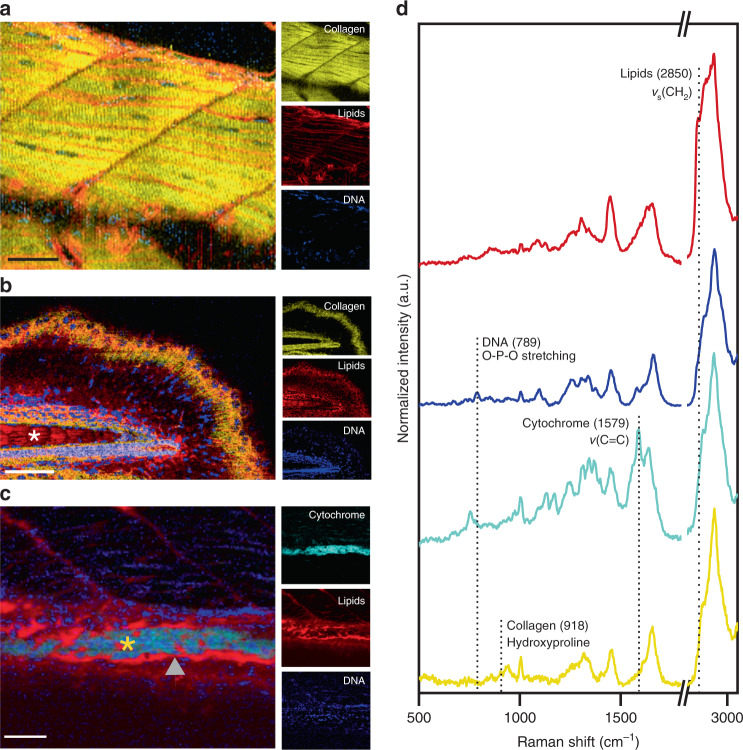
High-resolution confocal Raman spectroscopic imaging for anatomical tissue characterization. Confocal Raman spectroscopic imaging (cRSI) was used to image tissue regions of interest at 3 days post fertilization, with a spatial resolution of 0.5–1 µm. Univariate analysis was performed by integrating over a wavenumber range corresponding to relevant biomolecules: collagen-rich regions at 918 ± 20 cm^−1^ (shown in yellow), DNA-rich regions at 789 ± 10 cm^−1^ (shown in blue), lipid-rich regions at 2850 ± 10 cm^−1^ (shown in red), cytochrome-rich regions at 1579 ± 15 cm^−1^ (shown in cyan). **a** A representative image of the dorsal muscle tissue, from three independent cRSI scans (*N* = 3). Scale bar: 40 µm. **b** An exemplar scan of the tail tissue of a single embryo (*N* = 1), with the white asterisk indicating the notochord. Scale bar: 50 µm. **c** An exemplar scan of the developing gut of a single embryo (*N* = 1), with the orange asterisk marking the gut lumen and the grey triangle points indicating the gut lining. Scale bar: 40 µm. **d** Exemplar Raman spectra obtained from regions of intense univariate signal, with the labeled peaks used for univariate analysis.

We next investigated whether high-resolution cRSI could be used to resolve microscale tissue features in zebrafish embryos. We scanned a region of dorsal muscle tissue with a lateral resolution of 1 µm and performed univariate analysis to identify lipid-rich regions using a peak centered at 2850 cm^−1^ (*ν*_as_ CH_2_), DNA-rich regions using a peak centered at 789 cm^−1^ (O-P-O stretching), and collagen-rich regions using a peak centered at 918 cm^−1^ (hydroxyproline)^32^ (Fig. 3a). At this resolution, we were able to visualize the fine stratification of myofibers and muscle cells running along the anteroposterior axis. The individual muscle bundles were highly collagenous and interspersed with elongated lipid-rich regions. We attributed these lipid-rich regions to membrane phospholipids of muscle cells, due to the expected high density of lipid and the similarity in appearance to fluorescently stained muscle cell membranes^39^. This was supported by the observation of DNA-rich regions localized within individual nuclei running throughout the length of the muscle cells. It should be noted that peaks indicating the presence of lipids were weakly detected within the muscle fibers, which could potentially correspond to the presence of submicron intracellular lipid droplets that are present in muscle cells^40^.

Collagen-rich fibers could also be observed in the tail tissue, where they exhibited a distinct radial orientation (Fig. 3b). Individual nuclei were also clearly resolved throughout the tail. We used cRSI at a resolution of 0.5 µm to examine the embryonic gut, 3 days post-fertilization (Fig. 3c). Zebrafish guts undergo rapid development between 1 and 5 days post-fertilization, after which point the embryo is capable of external feeding^41,42^. Using cRSI, we were able to visualize the tract lining using a lipid peak signal centered at 2885 cm^−1^ (*ν*_s_ CH_3_), while the tract itself was rich in DNA, cytochrome, and collagen (Fig. 3d). Taken together, these examples demonstrate that cRSI can be used to obtain highly resolved biomolecular maps of zebrafish embryos, and that univariate analysis can be used to highlight clear distinctions between different tissues. These anatomical maps provide guidance for further cRSI studies, namely: (i) autofluorescence in the head and yolk sac means that Raman imaging is most optimal in the tail and gut regions, (ii) pigment interference is highest on the surface of the embryo, compared to deeper in the tissue, (iii) although a reduction in signal intensity was observed deep into the tissue (100 µm), high-quality spectra could still be obtained.

### Volumetric biomolecular profiling of mycobacterial infections

The developed zebrafish imaging protocol offered the opportunity to interrogate metabolic heterogeneities that are known to be intrinsically linked with variations in disease progression and drug susceptibility. For instance, current tuberculosis therapies involve a course of four drugs administered over a minimum of six months^43^; this is necessary as mycobacterial infections have a diverse biomolecular profile that make them a moving target during therapy. The design of improved therapies for treating mycobacterial infections requires an increased understanding of metabolic heterogeneity, and accordingly, we sought to demonstrate how volumetric cRSI could be used for biomolecular profiling of tuberculosis. We used an established zebrafish model in which embryos were injected with *Mycobacterium marinum* in the trunk at 2 days post-fertilization^44–46^. This injection route creates a localized infection that develops into a granuloma, the hallmark of tuberculosis. Indeed, at 4 days post-fertilization, we observed clear mycobacterial lesions in all infected embryos.

**Fig. 4:**
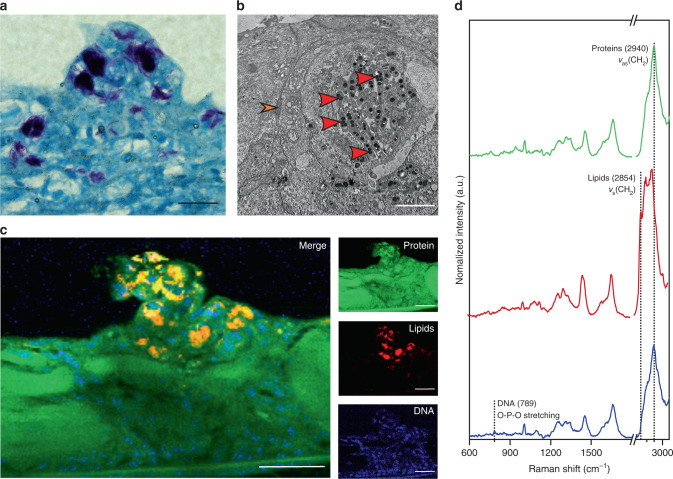
Confocal Raman spectroscopic imaging analysis of zebrafish embryo model of tuberculosis. An established model was used in which zebrafish embryos were injected with *M. marinum* to form localized mycobacterial lesions. All analysis was performed at 4 days post injection. **a** The model was verified by staining the lesions with a Ziehl–Neelsen stain, highlighting acid-fast mycobacteria (regions of purple), with a representative image selected from three independent biological replicates (*N* = 3). Scale bar: 20 µm. **b** Transmission electron micrograph of a mycobacterial lesion, a representative image of two independent biological replicates (*N* = 2). Osmium staining revealed the presence of mycobacteria (red arrows) with dense lipid clusters (stained black). The plasma membrane of the host cell is also visible (orange arrow). Scale bar: 2 µm. **c** Confocal Raman spectroscopic imaging (cRSI) was used to image mycobacterial lesions. The image shown is representative of four independent biological replicates (*N* = 4). Univariate analysis was performed by integrating over a wavenumber range corresponding to relevant biomolecules: protein-rich regions at 2940 ± 16 cm^−1^ (shown in green), lipid-rich regions at 2854 ± 10 cm^−1^ (shown in red), DNA-rich regions at 789 ± 10 cm^−1^ (shown in blue). Scale bars: 40 µm. **d** Representative spectra obtained from cRSI.

Before performing cRSI, we sought to validate and characterize this model using a complementary histological analysis. Localized mycobacterial clusters were first identified in embryo tissue sections using Ziehl–Neelsen acid-fast staining (Fig. 4a). The Ziehl–Neelsen stain is retained within mycobacteria, which exhibit acid-fastness due to the high mycolic acid content present within their bacterial cell walls. Mycolic acids are α-alkyl-branched β-hydroxylated fatty acids with hydrocarbon chains containing between 60–90 carbon atoms^47^. The mycolic acids give mycobacteria a lipid-rich Raman spectral profile, a feature that has been reported for 26 mycobacterial species (including *M. marinum*) and used to distinguish mycobacteria from other bacterial genera^48^. To further characterize the infection, we inspected the mycobacterial lesions using transmission electron microscopy (TEM) (Fig. 4b). This ultrastructural analysis revealed clustered, intracellular mycobacteria containing a number of lipid droplets, a feature that is typical of mycobacteria during infection^49–51^.

Having characterized the infection model and identified key Raman indicators for *M. marinum*, we used high-resolution cRSI scans across the infection site to provide clear, high-contrast images of mycobacterial lesions (Fig. 4c). Univariate analysis was able to readily identify distinct mycobacterial clusters as protein-rich regions using peaks centered at 1004 and 1665 cm^−1^ (*ν*_s_(C–C) and *ν*(C=O) respectively), and DNA-rich regions using peaks centered at 789 cm^−1^ (O-P-O stretching) and 1581 cm^−1^ (pyrimidine ring)^30,31^. These clusters also exhibited a set of elevated and sharpened peaks indicating the presence of lipid and hydrocarbon chains at 1081 cm^−1^ (*ν*(C–O) and *ν*(C–C))^28^, 1305 cm^−1^ (CH_2_ twisting), 1450 cm^−1^ (CH_2_ bending), 2850 cm^−1^ (*ν*_s_(CH_2_)) and 2885 cm^−1^ (*ν*_s_ CH_3_). We compared these spectra to corresponding measurements performed on liquid cultures of *M. marinum* collected at different growth stages (Fig. 4d). Both the clusters in the lesion and the liquid-cultured mycobacteria exhibited the characteristic Raman spectra that have previously been reported for mycobacteria with peaks at 1081 cm^−1^ (*ν*(C–O) and *ν*(C–C))^28^, 1305 cm^−1^ (CH_2_ twisting), 1450 cm^−1^ (CH_2_ bending), 2850 (*ν*_s_ CH_2_) and 2885 cm^−1^ (*ν*_s_ CH_3_)^48^ that were strongly elevated compared to the signal observed in the surrounding zebrafish tissue (Supplementary Fig. 2a). We also observed subtle changes in the spectral profile of the liquid-culture *M. marinum* corresponding to their growth stage (Supplementary Fig. 2b). Similar observations have been reported for other mycobacterial species and these changes are thought to reflect the metabolic changes that occur as the mycobacteria shifts from logarithmic to stationary growth^52^. We did, however, observe spectral differences between the liquid-culture *M. marinum* and the clusters observed in the lesion. This observation was unsurprising given the stark difference between the two environments (i.e. a nutrient-rich medium versus a hostile host).

**Fig. 5:**
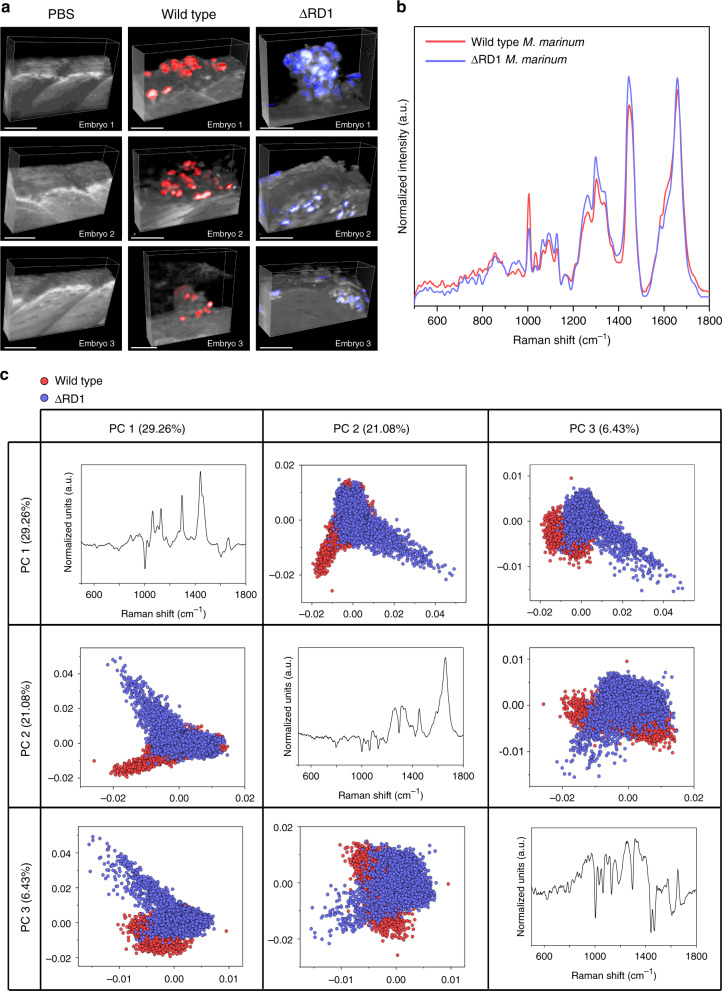
Biomolecular profiling and discrimination of mycobacterial infections. **a** Volumetric confocal Raman spectroscopic imaging (cRSI) of zebrafish embryos infected with either wild type or ∆RD1 *M. marinum* or injected with PBS as a negative control. cRSI was performed at 4 days post injection, and 3D reconstructions show embryo tissue in gray with mycobacterial clusters displayed in red (wild type) or blue (∆RD1). Scale bars: 50 µm. **b** Mean spectra of the mycobacterial clusters extracted from the volumetric cRSI scans for wild type *M. marinum* (red trace) and the ∆RD1 mutant bacteria (blue trace). **c** Principal component analysis showed clear separation of the wild type *M. marinum* (red markers) and the ∆RD1 mutant bacteria (blue markers) using the three principal components (PC1, PC2, PC3).

These results collectively demonstrate that Raman spectroscopy can be used to detect subtle metabolic differences in *M. marinum* and that cRSI can be used to identify mycobacterial clusters present in infected zebrafish embryos. With this knowledge, we sought to demonstrate how cRSI could be used for comprehensive biomolecular profiling of mycobacterial infections and discrimination between lesions arising from different *M. marinum* strains. Using the same infection model, we injected zebrafish embryos with either wild type *M. marinum* or a mutant strain (∆RD1). This mutant strain lacks the RD1 locus, which contains genes for several important antigens secreted by *M. marinum* at the early stage of infection that are used by the mycobacteria to manipulate the host environment^53^. We then performed volumetric cRSI scans across mycobacterial lesions generated by either the wild type or the ∆RD1 *M. marinum* (Fig. 5a). In order to make relevant spectral comparisons between the two groups, we developed a computational approach that could objectively identify mycobacterial clusters in different lesions. This process applied a threshold based on the most prominent *M. marinum* peaks to identify mycobacterial clusters and then collected spectral information from each cluster-associated voxel into a matrix for biomolecular profiling (see Supporting Information). The mean-averaged Raman spectra extracted from the mycobacterial clusters in the lesions generated by wild type and ∆RD1 *M. marinum* had a similar overall composition but exhibited several differences in the relative peak intensities. Notable differences were observed in DNA/RNA regions at 750 cm^−1^ (ring breathing mode of DNA/RNA bases) and 789 cm^−1^ (O-P-O stretching), as well as in peaks associated with mycolic acid at 1087 cm^−1^ (*ν*(C–C)), 1305 cm^−1^ (CH_2_ twisting) and 1450 cm^−1^ (CH_2_ bending)^52^. (Fig. 5b).

These spectral differences enabled the two groups to be clearly separated using principal component analysis (PCA), an unsupervised method of dimensionality reduction (Fig. 5c). Inspecting the principal components, we observed that the main source of variation between the two populations was in the vibrations corresponding to lipids, with sharpened peaks at 1065 cm^−1^ ((C–C)-stretching), 1128 cm^−1^ (*ν*(C–C)), 1298 cm^−1^ (acyl chains), 1439 cm^−1^ ((C–C)-bending), 1450 cm^−1^ (CH_2_ bending). Notably, the first principal component (PC1) had a strong contribution from saturated fatty acids (e.g., stearic acids or palmitic acids), which was evidenced by characteristic molecular vibrations at 1065 cm^−1^ (C–C stretching), 1128 cm^−1^ (*ν*(C–C)), 1298 cm^−1^ (acyl chains), and 1439 cm^−1^ (C–C bending). This suggested that the clusters containing ∆RD1 mutants had an increased presence of saturated fatty acids compared to the wild type group. This is an intriguing observation because it is well established that mycobacterial lipid metabolism has a major impact on the course of infection^54^. Moreover, the second principal component (PC2) suggested variations in both DNA at 789 cm^−1^ (O-P-O stretching) and saturated lipids 1650 cm^−1^ (*ν*(C=C)). However, it should be noted that PC2 separated the two bacterial strains less effectively and could thus have reflected sample variation in the study. We then quantified the separation between the two bacterial populations using partial least-squares discriminant analysis (PLS-DA). Using this supervised method, we measured a near-complete separation (99.7%) between the wild type and ΔRD1 mutant groups using three latent variables and a Venetian blinds cross-validation with ten data splits (Supplementary Fig. 3).

These results demonstrate that cRSI can not only be used to create volumetric images of mycobacterial lesions but that positional and compositional data can be extracted and analyzed to generate biomolecular profiles that can discriminate infections arising from different *M. marinum* strains. This represents an unprecedented level of biomolecular analysis in zebrafish embryos. The precise biomolecular features underpinning the spectral separation, which could relate to a number of factors in mycobacterial pathogenesis, is subject to an ongoing investigation.

### In vivo time-lapse imaging of wound response

Finally, we sought to develop a cRSI protocol for in vivo, time-lapse imaging of zebrafish embryos. This method would allow hyperspectral and time-lapse biomolecular imaging in a living animal model. Near-infrared laser sources offer minimal photodamage and high compatibility with living tissue, therefore, we elected to build our in vivo cRSI method using a 785 nm laser. To verify the biocompatibility of this approach, we performed a series of three cross-sectional cRSI scans of living zebrafish embryos, which we then compared to unexposed controls. These cross-sections were set up to traverse through the entire dorsoventral axis of the embryos, including muscle tissue and the caudal artery and vein. After each scan, the scanned embryos were compared to unscanned controls using bright field microscopy, with no visible differences observed (Supplementary Fig. 4a, b). We also collected bright field videos showing the zebrafish embryo heartbeat, and caudal artery and vein, before and after each of the three Raman scans. There was no visible effect on the blood flow and no significant changes in the quantified heart rate (Supplementary Fig. 4c, Supplementary Movies 1 & 2).

Furthermore, the Raman spectra collected during the scans showed no characteristic signs of damage, such as increased tissue fluorescence or the presence of carbon peaks typical of laser-induced burning (Supplementary Fig. 4d). We performed a PCA on all spectra collected during the live-embryo imaging to identify changes in the Raman spectra that would indicate an effect on the scanned tissue. We observed no such changes; indeed, there was no identifiable separation between the three consecutive scans in all the embryos tested (Supplementary Fig. 5). Although a full study is required to test for more subtle biological responses, such as inflammation, these results suggested that cRSI using a 785 nm laser did not cause any tissue damage or changes to circulation. We thus proceeded to test this laser for in vivo cRSI by imaging a larger area of a living zebrafish embryo (Supplementary Fig. 6a). We obtained well-resolved Raman spectra, with clearly identifiable peaks corresponding to DNA at 789 cm^−1^ (O-P-O stretching), lipids at 1450 cm^−1^ (CH_2_ bending), proteins at 1004 cm^−1^ (*ν*_s_(C-C) and collagen-associated amide I at 1665 cm^−1^ *ν*(C=O) (Supplementary Fig. 6b).

**Fig. 6:**
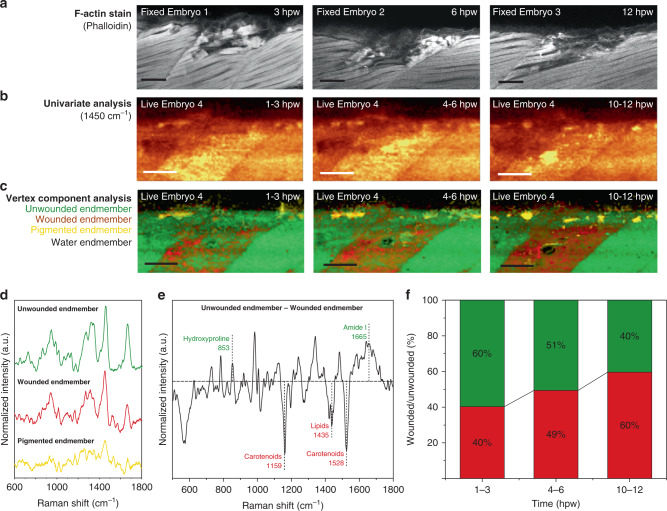
cRSI of living zebrafish embryo wound response. Living zebrafish were imaged for the 12 h following a controlled stab wound performed at 3 days post fertilization. **a** Confocal fluorescence microscopy of F-actin was highlighted by phalloidin staining (red) at 3, 6 and 12 hours post wounding (hpw) on three independent biological replicates at each time point (*N* = 3). **b** Univariate analysis of the confocal Raman spectroscopic imaging (cRSI) scans of a living zebrafish embryo at 1–3, 4–6 and 10–12 hpw. Univariate analysis was performed by integrating over a wavenumber range at 1450 ± 30 cm^−1^. **c** The tissue damage in these images was clearly visualized by using multivariate vertex component analysis (VCA), with the wounded component displayed in red and the unwounded component displayed in green. A water component (black) and a pigment component (yellow) were also identified. Scale bars: 40 µm. **d** The three tissue endmembers identified by VCA (the water endmember spectra not shown). **e** Analysis of the unwounded and wounded endmembers identified by the VCA. The difference spectrum when the wounded endmember was subtracted from the unwounded endmember. The horizontal dashed line indicates zero difference between the two endmembers. The dotted vertical lines indicate the center of the annotated peaks that were clearly different between wounded and unwounded tissue, with the color coding indicating in which component they were dominant. Green indicates higher presence in the unwounded component and red indicates higher presence in the wounded component. **f** Relative abundance of wounded and unwounded component in the injured somite at the three time points extracted from pixel intensity histograms produced by the VCA. The wounded component is shown in red and the unwounded component is shown in green. Figures 6b–f are a representative set of images and analysis taken from three independent biological replicates (*N* = 3).

Having verified the compatibility and effectiveness of the 785 nm laser, we sought to apply in vivo cRSI for biomolecular analysis in an established zebrafish embryo wound model. We created a controlled and reproducible wound by making a small incision in the epaxial myotome using a surgical tungsten needle (see methods). This procedure produced a clearly visible wound, with damaged muscle filaments visible by phalloidin staining of F-actin (Fig. 6a). We followed the wound response in a living embryo using cRSI, performing three 2 h scans over a total period of 12 h (Fig. 6b), and then performed vertex component analysis (VCA) on the time-lapse images. This multivariate analysis identified four endmembers corresponding to wounded tissue, unwounded tissue, water, and pigment (Fig. 6c, d, Supplementary Fig. 7). For each timepoint, the water component was the primary external feature, the unwounded tissue component dominated the somites adjacent to the wound site, while the pigment component was present on the embryo surface, as observed previously. Comparing the wounded and unwounded components revealed several biomolecular changes associated with the wound response (Fig. 6e). The wounded component exhibited an increased presence of carotenoid pigments at 1159 and 1528 cm^−1^ (conjugated C–C and C=C stretch) and lipids at 1435 cm^−1^ (CH_2_ scissoring). Conversely, there was a reduction in peaks at 852 cm^−1^ (hydroxyproline), 918 cm^−1^ (hydroxyproline), 1246 cm^−1^ (*ν*_s_(C–N)), and 1665 cm^−1^ (amide I), potentially indicating a reduction in collagen content in the wounded component. Moreover, the wounded tissue component increased in area and intensity over time to become the dominant component in the wounded somite after 12 h (Fig. 6e). These biomolecular changes can be correlated to the loss of muscle tissue, clearly seen in the phalloidin stains. The precise biomolecular mechanisms responsible for these observations are subject to an ongoing investigation, however, this demonstration illustrates how cRSI can be used for repeated in vivo biomolecular imaging in living zebrafish embryos.

## Discussion

In this report, we demonstrate that cRSI can be used as a versatile, label-free methodology for analytical biomolecular imaging in zebrafish embryos. We use cRSI to visualize fine structural details, such as muscle fibers or individual nuclei, and to generate full volumetric biomolecular maps of whole zebrafish embryos. We demonstrate further applicability of cRSI by generating volumetric images of mycobacterial lesions in a zebrafish embryo model of tuberculosis, showing that PCA and PLS-DA could be used to discriminate between zebrafish embryos infected with either wild type or ΔRD1-mutant *M. marinum*. We also showed that cRSI could be used as an in vivo imaging tool in living zebrafish embryos. We recorded time-lapse scans of wounded tissue and used VCA to locate the wound site and identify temporal biomolecular changes occurring during the acute wound response. While cRSI does not have the high specificity of immunostaining and has lengthy scan times that prevent temporal analysis of rapid biological processes, the ability to perform volumetric and in vivo imaging in unlabeled embryos should provide a host of new opportunities for zebrafish research that can readily complement existing fluorescence imaging techniques. Specifically, we anticipate that cRSI could be used for correlative fluorescence and Raman spectroscopic imaging. A correlated approach was described by Bennet et al., who performed fluorescence imaging and Raman point spectra collection on the same embryo^20^. Extending this correlative imaging platform to include both fluorescence imaging and cRSI would not only enable more targeted cRSI imaging, but also allow for more refined biomolecular profiling. For example, cRSI analysis could be correlated with the expression of specific fluorescent markers, such as those indicating different stages of the bacterial replication cycle during infection. Furthermore, cRSI can also complement other biomolecular analyses, such as transcriptomics, proteomics, lipidomics, and metabolomics, in order to decipher the significance of biomolecular variations in development and disease. Moreover, we anticipate that further development of other Raman spectroscopic imaging strategies, such as light sheet Raman^24^ or nonlinear Raman imaging^21,22^, will be instrumental to fully integrate Raman spectroscopic imaging in the zebrafish field.

## Methods

### Ethics statements

Experiments involving zebrafish were conducted in accordance with UK Home Office requirements (Animals Scientific Procedures Act 1986, project licence P5D71E9B0). All experiments were conducted up to 5 days post-fertilization, except for *M. marinum* experiments, where fish were kept until 6 days post-fertilization according to (Project licenses: PPL P84A89400 and P4E664E3C).

### Zebrafish husbandry

Transparent *TraNac* mutant fish were obtained from Julian Lewis, London Research Institute, London. Fish were kept in the CBS facility of Imperial College London and were reared and maintained according to standard practices at 28.5 °C on a 14-h light/10-hour dark cycle. Embryos were raised in E2 water^55^ supplemented with 0.3 ppm methylene blue^55^. Embryos were kept in Petri dishes at a density of ~50 embryos per dish and E2 water was replaced daily.

### cRSI anatomical imaging

Zebrafish embryos at 3 days post-fertilization were anesthetized in 4.2% (w/v) MS222 and fixed overnight in 4% (w/v) paraformaldehyde. The fixed embryos were mounted on a glass slide in 0.8% (w/v) low melting point agarose (NuSieve^TM^ GTG^TM^ Agarose, Lonza). During mounting, the embryos were gently aligned to ensure the sagittal plane was parallel with the glass slide. The mounted samples were placed in 10 cm Petri dishes and immersed in ~50 mL of E2 water, in order to dissipate the heat from the laser and avoid melting of the agarose gel. E2 water was used to maintain an osmotic balance and avoid swelling or contraction of the mounted embryo. Embryos were imaged using a confocal Raman microscope (alpha300R+, WITec, GmbH, Germany) with a 532 nm laser light source at 35 mW power output applied through a ×63/1.0 NA water-immersion objective lens (W Plan-Apochromat, Zeiss, Germany). Inelastically scattered light was collected through the objective lens and directed via a 100 μm diameter silica fiber, acting as a confocal pinhole, to a high-throughput imaging spectrograph (UHTS 300, WITec, GmbH, Germany) with a 600 groove/mm grating and equipped with a thermoelectrically cooled (−60 °C) back-illuminated charge-coupled device camera.

3D whole-embryo Raman images were acquired with a spectral resolution of ∼11 cm^−1^. All 3D images were measured with a 10 µm lateral and 10 µm axial spatial resolution. With an integration time of 0.5 s per spectrum, the scan time was ~4 h per image. The entire 3D stack consisted of 11 images and was collected over a period of 48 h. High-resolution tissue imaging was performed using the same procedure but with a lateral spatial resolution of 0.5 or 1.0 µm, as stated. Spectral preprocessing was performed using WITec ProjectFOUR software. Briefly, spectra were first cropped to remove Rayleigh scattered light. Background subtraction was an optional step that was performed here to avoid the signal intensity being skewed by autofluorescence, which varied between different zebrafish embryos. This was performed using a “shape” background filter with a parameter size of 500 and noise factor of 0 to efficiently remove tissue autofluorescence (Supplementary Fig. 8). Finally, each Raman spectra were normalized to the area under the curve and univariate analysis was performed by integrating over a wavenumber range corresponding to relevant peaks: protein-rich regions (2940 ± 16 cm^−1^), lipid-rich regions (2850 ± 5 cm^−1^), carotenoid-rich regions (1159 ± 16 cm^−1^), collagen-rich regions (918 ± 20 cm^−1^), DNA-rich regions (789 ± 10 cm^−1^), lipid-rich regions (2885 ± 10 cm^−1^), and cytochrome-rich regions (1579 ± 15 cm^−1^). It should be noted that extended imaging periods may result in drift, which could negatively affect the final image. Although we did not observe or correct for any drift in these studies, it is possible to apply drift correction algorithms. One such strategy is to use a feature detector to detect image drift over several images and then apply, for example, M-estimator SAmple and Consensus (MSAC) to estimate the geometric transform between subsequent images and thus perform drift correction.

### Zebrafish embryo model of tuberculosis

*M. marinum* from a glycerol stock were cultured on Middlebrook 7H10 agar plates (BD Biosciences) supplemented with oleate-albumin-dextrose-catalase (OADC) enrichment (BD Biosciences). Plates were incubated at 28.5 °C for 7–10 days. *M. marinum* from developed colonies were collected and resuspended in 10 mL of Middlebrook 7H9 medium (BD Biosciences) supplemented with albumin-dextrose-catalase (ADC) enrichment (BD Biosciences), to an optical density at 600 nm (OD_600_) of 0.1. Cultures were incubated statically at 28.5 °C for 24–36 h to reach an OD_600_ of 0.5–0.8. Bacteria were then spun down, washed in PBS and resuspended to the desired concentration in 2% (w/v) polyvinylpyrrolidone in phosphate buffered saline (PVP/PBS, Sigma Aldrich)^56^. 1 nL of this solution, containing ~50 CFU of wild type *M. marinum* or 150 CFU of ΔRD1 *M. marinum*, were injected into the trunk of zebrafish embryos at 2 days post-fertilization^46^. The injected embryos were then kept in 10 cm diameter petri dishes at a density of 20 embryos per dish at 28 °C until 4 days post injection when they were anesthetized in 4.2% (w/v) MS222 and then fixed for histology, electron microscopy, or cRSI.

### Histology of mycobacterial lesions

Zebrafish embryos infected with wild type *M. marinum* were fixed overnight in 4% (w/v) paraformaldehyde and then embedded in paraffin wax, and sectioned onto Thermo Scientific™ SuperFrost Plus™ adhesion slides (Fisher Scientific) (section thickness of 4 µm). The sections were stained with Ziehl–Neelsen stain to visualize the *M. marinum*. Briefly, the sections were dewaxed and rinsed first in ethanol and then in deionized water. The sections were then transferred to carbol fuchsin solution at 60 °C for 30 min. The sections were then washed in acid alcohol solution containing 1% (v/v) hydrochloric acid in 70% (v/v) ethanol until there was no stain observed coming off of the sections. The sections were then counterstained for ~1 min in 0.25% (v/v) methylene blue in 1% (v/v) acetic acid. The sections were then dehydrated, mounted, and imaged using a Zeiss Axio Observer microscope.

### Electron microscopy of mycobacterial lesions

Zebrafish embryos infected with wild type *M. marinum* were fixed overnight in 1% (w/v) glutaraldehyde (Electron Microscopy Sciences) in 60 mM HEPES (Gibco). These embryos were post-fixed in 2% (w/v) aqueous osmium tetroxide (Electron Microscopy Sciences) for 1 h at room temperature, followed by 1 h staining with 1% (w/v) tannic acid (Sigma Aldrich). The embryos were then dehydrated using sequential 2 × 10 min incubations in 70, 80, 90, and 100% (v/v) ethanol. The embryos were embedded in Quetol 651 (TAAB Laboratories Equipment) in a flat mold and oriented so that the lesion was clearly visible through the resin, which was then cured at 60–65 °C for 40 h. The resin block was manually trimmed using a razor blade and then sectioned using an ultramicrotome (RMC PowerTome) equipped with a glass knife, with an automatic advancing distance of 1 µm per section. The sections were collected onto a glass slide, stained with Toluidine Blue, and observed under a light microscope. This allowed the identification of sections containing the mycobacterial lesion, which were further sectioned using a 45° diamond knife (Diatome). 60 nm sections were collected onto an ultra-thin Formvar 100 mesh copper grid (Electron microscopy Sciences), stained with UranyLess for 2 min and lead citrate (Electron Microscopy Sciences) for 1 min and then coated with a 4 nm layer of carbon using an evaporation coater (Low Vacuum Coater Leica EM ACE600). These stained sections were used for transmission electron microscopy using a Titan (FEI) 80/300 operating at 80 kV.

### cRSI of mycobacterial lesions

Zebrafish embryos infected with wild type or ΔRD1 *M. marinum* were fixed in 4% (w/v) PFA overnight and mounted in 0.8% (w/v) low melting point agarose, as described above. Single plane cRSI images were captured of lesions in the wild type group using a 532 nm laser at a spatial resolution of 1 µm. With an integration time of 1 s, the total scan time was ~4 h. 3D cRSI images were captured of lesions in both the wild type and ΔRD1 injected groups (*N* = 3) using a 532 nm laser with a lateral resolution of 2 µm, an axial resolution of 2 µm, and an integration time of 1 s. Depending on the sample size, the total scan time was 12–15 h. A negative control was performed in which volumetric cRSI images were captured of zebrafish embryos that were injected with resuspension buffer alone (2% (w/v) polyvinylpyrrolidone in PBS), rather than *M. marinum*.

The volumetric cRSI images were processed by spectral cropping and background subtraction, as described above, and finally analyzed using a custom MATLAB script (see Code Availability Statement). Briefly, thresholding was performed using the peak intensity between 2844–2864 cm^−1^ (identified as mycolic acids) with any Raman spectra and discarding any Raman spectra with a peak intensity below this threshold. Individual mycobacterial clusters were then identified using blob analysis, with each Raman spectrum assigned to its corresponding blob (bacterial cluster)^57^. Colored blob images were then imported into Icy (Icy—Open Source Image Processing Software) to generate merged images. Raman spectral variation of bacterial clusters was assessed using both PCA and PLS-DA of normalized (area under the curve), mean-centered Raman spectra. These analyses were conducted in PLS_Toolbox (Eigenvector Research) within the MATLAB environment. PLS-DA spectral classification was performed using three latent variables and a Venetian blinds cross-validation with 10 data splits.

### Raman spectroscopy of liquid-culture *M. marinum*

Samples of mycobacteria were collected at an OD_600_ of 0.6, 0.9, or 2.3, centrifuged and then fixed overnight in 4% (w/v) PFA at a concentration of OD_600_ = 0.5 per mL of PFA. The mycobacteria were transferred to a magnesium fluoride slide and Raman spectra were collected for five replicates of each mycobacteria using a 20x/0.4 NA air objective lens (EC Epiplan, Zeiss, Germany) and a 0.5 s integration time.

### Tolerance of living zebrafish embryo to cRSI

Raman imaging of live zebrafish embryos was performed using a 785 nm laser to reduce possible phototoxic effects^57^. To assess laser tolerance, zebrafish embryos at 3 days post-fertilization were anesthetized in 4.2% (w/v) MS222 and mounted onto a glass slide in 0.8% (w/v) low melting point agarose, as previously described, but with no fixation step. After gelation, the glass slide was transferred to a 10 cm diameter Petri dish containing E2 water supplemented with 4.2% (w/v) MS-222. cRSI was performed on a living zebrafish embryo using a 785 nm laser light source at 85 mW power output in a heat chamber set at 28.5 °C. Three Raman images were measured in sequence using a 1.5 s integration time with a 2 µm spatial resolution. Before and after the three scans, 30 s videos were recorded of the heartbeat using bright field microscopy and screen recording software (Icecream Apps). In parallel, an identical protocol was performed without Raman imaging, as a negative control (*N* = 4). We compared the difference in heart rate between the Raman scanned embryo and the unscanned embryo for each experimental repeat and at each timepoint. We performed a linear regression, with the slopes of each of these regression lines tested individually using a two-sided Wald test to determine significant deviation from zero. The whole set of slopes were then tested using a Wilcoxon signed-rank test. A three-component PCA was performed on background-subtracted and normalized Raman spectra across each timepoint and each zebrafish embryo. It should be noted that the live imaging with the 785 nm laser used a higher optical section, which resulted in background signal from the underlying substrate. We thus cropped the region containing spectral artifacts (917–1095 cm^−1^) to enable fair comparison during PCA.

### cRSI of living zebrafish embryo wound response

To assess wound response, zebrafish embryos at 3 days post-fertilization were anesthetized in 4.2% (w/v) MS222 and then mounted on a 2% (w/v) agar plate^55^. The embryos were wounded by making a single incision using a surgical tungsten needle (W20-148-01×2.0, World Precision Instruments) in the dorsal myotome opposite the anal pore. Immediately after wounding, the embryos were transferred to a Petri dish containing E2 water to allow recovery. Next, the wounded zebrafish embryos were covered in 0.8% (w/v) low melting point agarose at ~40 °C and aligned, as previously described. After gelation, the glass slide was transferred to a 10 cm Petri dish containing E2 water supplemented with 4.2% (w/v) MS-222. cRSI was performed on living zebrafish embryos using a 785 nm laser light source at 85 mW power output in a heat chamber set at 28.5 °C. Three planar scans were performed over a period of 12 h using a spatial resolution of 2 × 2 µm. With a 1.5 s integration time, each scan lasted ~2 h.

The Raman spectroscopy images were processed by spectral cropping and background subtraction, as described above, and then a weighted spectrum subtraction of a background pixel spectrum was used to remove the spectral contribution of the underlying substrate. An exemplar univariate image of the wounded region was then generated via a sum filter between 1435 and 1465 cm^−1^. Processed Raman spectra were analyzed using a custom MATLAB script (see Code Availability Statement). Briefly, sequential Raman spectroscopy images of the wounded region were concatenated for processing before normalization (area under the curve) and removal of spectra containing cosmic peaks. Vertex component analysis (VCA) was performed to identify 10 endmembers. A spectral comparison was made between these endmembers and a live zebrafish control, scanned using a 785 nm laser. This was used to identify the 4 most relevant endmembers, representing water, pigment, and two different tissue signatures. The selected endmembers were then used to perform non-negatively constrained least-squares regression to generate abundance images for each endmember. The abundance images associated with the two tissue components were further analyzed in ImageJ to quantitively compare the relative temporal changes in wound response. A region of interest corresponding to the wounded somite was defined and the area under the curve of was measured for the pixel intensity histograms corresponding to the two tissue components.

### F-actin staining of wounded zebrafish embryos

The stab wound model was verified by F-actin staining, using a protocol adapted from Goody et al.^58^, and performed on a separate set of embryos to those used in the Raman scans with different embryos used for each timepoint. The wounded embryos were fixed overnight in 4% (w/v) PFA at 4 °C. The next day, the embryos were washed 3 × 5 min in 0.5 mL 0.1% (v/v) Tween 20 (Sigma) in PBS (Gibco) and then washed for 90 min at room temperature in 0.5 mL 2% (v/v) Triton X-100 (Sigma) in PBS (Gibco). The Triton X-100 solution was then replaced with 19 µL of fresh 2% (v/v) Triton X-100 with 1 µL of rhodamine phalloidin (Thermo Fisher). Staining was performed overnight at 4 °C with gentle shaking. Prior to imaging, the embryos were gradually transferred to 80% (v/v) glycerol through subsequent washing steps of increasing glycerol concentration (20, 40, 60, 80%). The embryos were then mounted on a glass bottomed petri dish (Cellview Cell Culture Dish, PS 35/10 MM, glass Bottom, Greiner Bio-One) and then imaged using an SP5 inverted confocal fluorescence microscope (Leica) with a 20x/0.5 PL FLUOTAR objective lens (Leica).

### Data analysis

Microsoft Excel (version 16) and Origin (version 2018b) were used for all data processing and statistical analysis. MATLAB 2019a was used for data analysis.

### Reporting summary

Further information on research design is available in the Nature Research Reporting Summary linked to this article.

## Supplementary information

Supplementary Information

Description of Additional Supplementary Files

Supplementary Movie 1

Supplementary Movie 2

Reporting Summary

## Data Availability

Raw data are available online at 10.5281/zenodo.4059924.

## Code availability

MATLAB codes used for analysis in this paper is available at: https://github.com/conor-horgan/Raman-Zebrafish-Analysis
