# Supplementary material for: In vivo biomolecular imaging of zebrafish embryos using confocal Raman spectroscopy
Source: Nat Commun. 2020 Dec 2;11:6172. doi: 10.1038/s41467-020-19827-1 (PMC7710741; doi:10.1038/s41467-020-19827-1)
Supplement: Supplementary file 1 — Supplementary Information [file 41467_2020_19827_MOESM1_ESM.pdf]

In Vivo Biomolecular Imaging of Zebrafish Embryos using Confocal Raman Spectroscopy

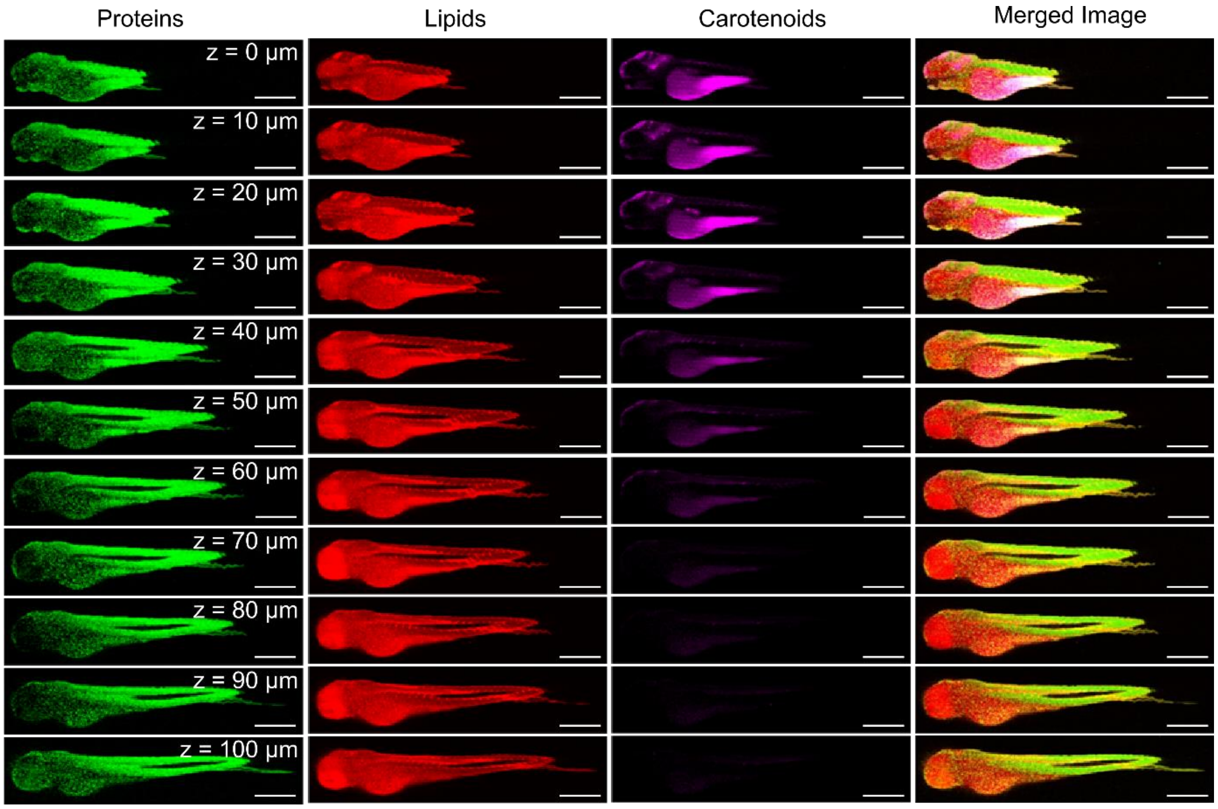

Supplementary Figure 1: Volumetric confocal Raman spectroscopic imaging of whole zebrafish embryos. Confocal Raman spectroscopic imaging (cRSI) was used to image a fixed embryo (N = 1) at 3 days post fertilization at 10 µm in-plane resolution and 10 µm out-of-plane resolution. This figure shows the entire stack collected from the cRSI scan with univariate normalized peak intensity. The Z-position relative to the top slice of the stack is indicated. Z = 0 µm, 30 µm, 60 µm and 90 µm is shown in Figure 1. Scale bars: 500 µm. Univariate analysis was performed by integrating over a wavenumber range corresponding to relevant biomolecules: protein-rich regions at 2940 ± 16 cm^{-1} (shown in green), lipid-rich regions at 2850 ± 5 cm^{-1} (shown in red), carotenoid-rich regions at 1159 ± 16 cm^{-1} (shown in magenta).

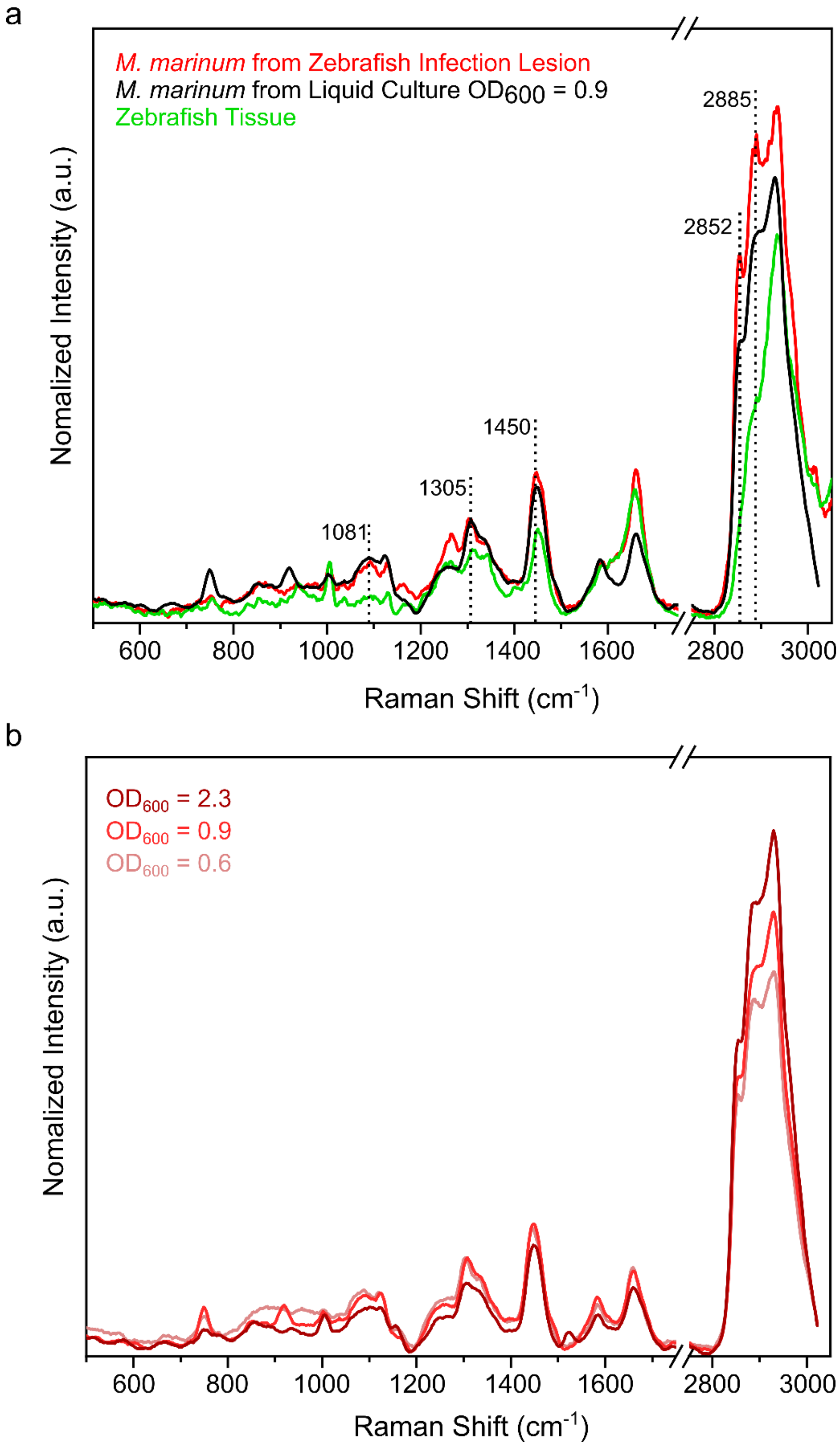

Supplementary Figure 2: Comparison of Raman spectra from zebrafish infection model and bacterial culture. a) Raman spectra of *M. marinum* clusters in a lesion of an infected zebrafish embryo (red line), the zebrafish tissue surrounding the lesion (green line) and *M. marinum* cultured *in vitro* at an optical density at 600 nm (OD_{600}) of 0.9. The spectra displayed were obtained from confocal Raman spectroscopic imaging (cRSI) of the *M. marinum* lesion (red trace, representative spectrum of ~500 pixels) and the surrounding zebrafish tissue (green trace, representative spectrum of ~6000 pixels) at 4 days post infection. For comparison, a Raman spectra of *in vitro* cultured *M. marinum* at an OD_{600} of 0.9

(mean spectrum of 9 consecutive measurements). Important peaks associated with mycobacterial mycolic acids are indicated with dotted lines. **b)** Raman spectra from *M. marinum* collected at different stages of liquid culture: OD_{600} = 2.3 (dark red line), OD_{600} = 0.9 (red line), and OD_{600} = 0.6 (pink line). Data shown are mean average spectra from 5, 9 and 6 measurements, respectively.

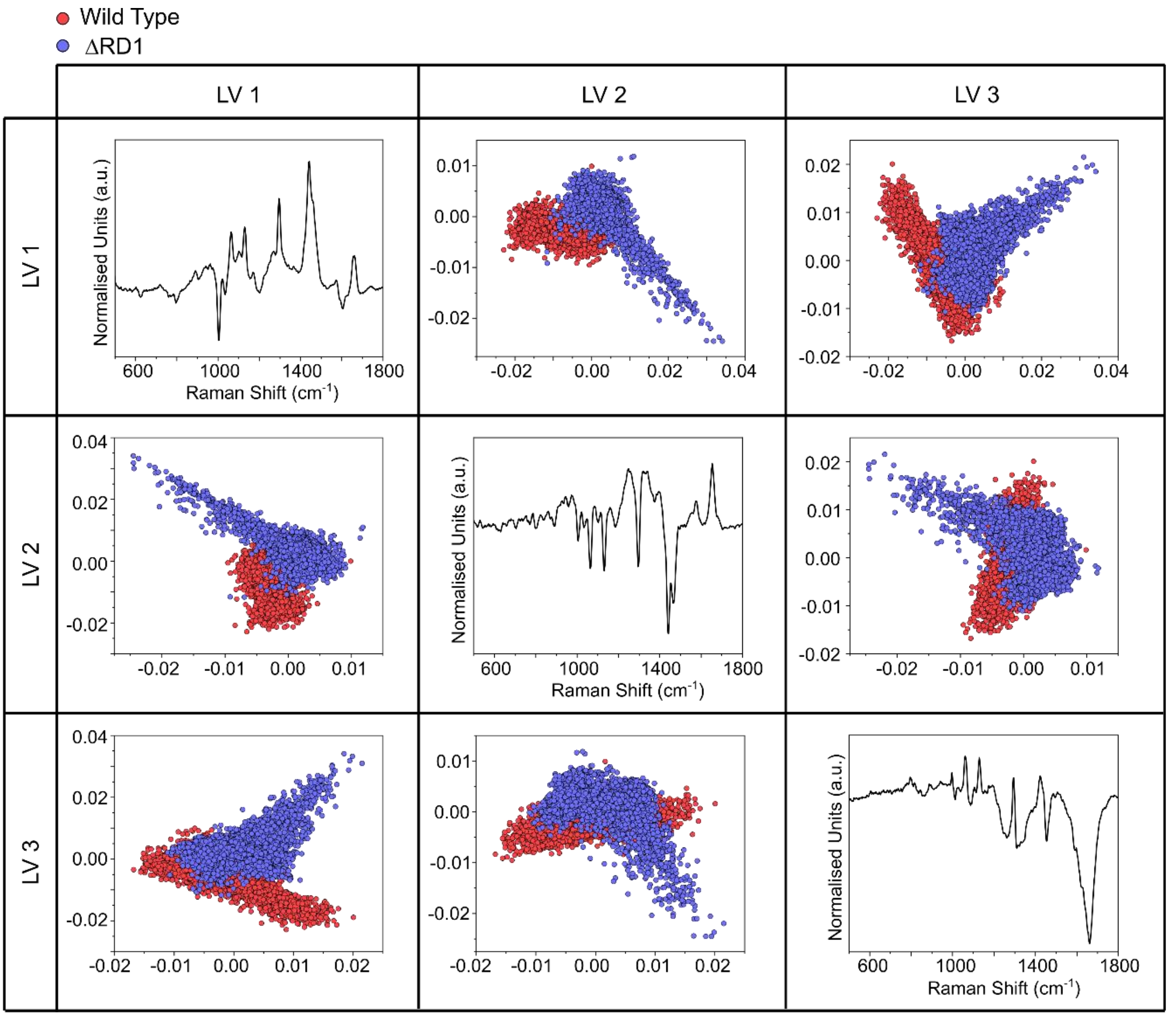

Supplementary Figure 3: Comparison of wild type and ΔRD1 mutant *M. marinum*. Partial least squares discriminant analysis of the lesions formed from injecting zebrafish embryos with wild type *M. marinum* (red markers) or ΔRD1 mutant *M. marinum* (blue markers), with spectra of the identified latent variables (LV1, LV2, LV3).

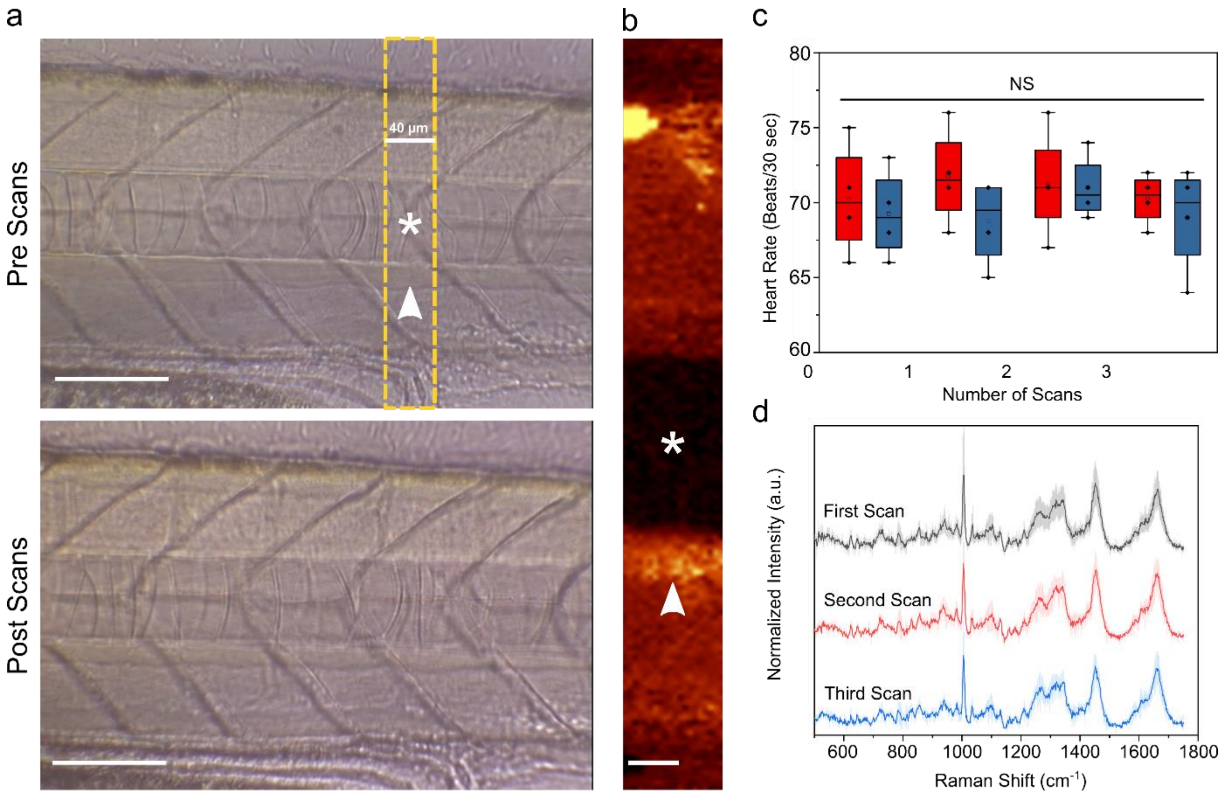

Supplementary Figure 4: Tolerance of living zebrafish embryos to confocal Raman spectroscopic imaging. **a)** Representative bright field images of the zebrafish embryo before and after three planar confocal Raman spectroscopic imaging (cRSI) scans, performed on four independent embryos (N = 4). These images showed no visible signs of laser damage. The yellow dashed box indicates the scan area which extended over the entire dorsoventral axis at width of 40 µm. The asterisk indicates the notochord and the arrowhead indicates the caudal artery. Scale bars: 100 µm. **b)** Representative cRSI scan performed on one of the four scanned zebrafish embryos (N = 4). The intensity in these images were generated by univariate analysis performed by integrating over a wavenumber range of 1665 ± 20 cm^{-1} (amide I) and highlight the zebrafish tissue. The asterisk indicates the notochord and the arrowhead indicates the caudal artery. Scale bar: 20 µm. **c)** Mean heart rate of zebrafish embryos measured before the study and after each cRSI scan (blue), with data generated from four independent zebrafish embryos (N = 4). This analysis showed no significant difference with unscanned controls (red). Lines indicates median, box boundaries indicate 1st and 3rd quartile and whiskers indicate maximum and minimum values. Data shown with statistical comparison performed using a regression analysis between cRSI scanned and unscanned control fish followed by a two-sided Wilcoxon signed-rank test to test for divergence over time. No significant divergence was observed ($p = 0.715$). **d)** Mean Raman spectra collected from four independent zebrafish embryos scanned with cRSI (N = 4). Data shown is mean ± standard deviation after the first scan (black line, gray error bars), the second scan (red line, pink error bars), and the third scan (blue line, light blue error bars).

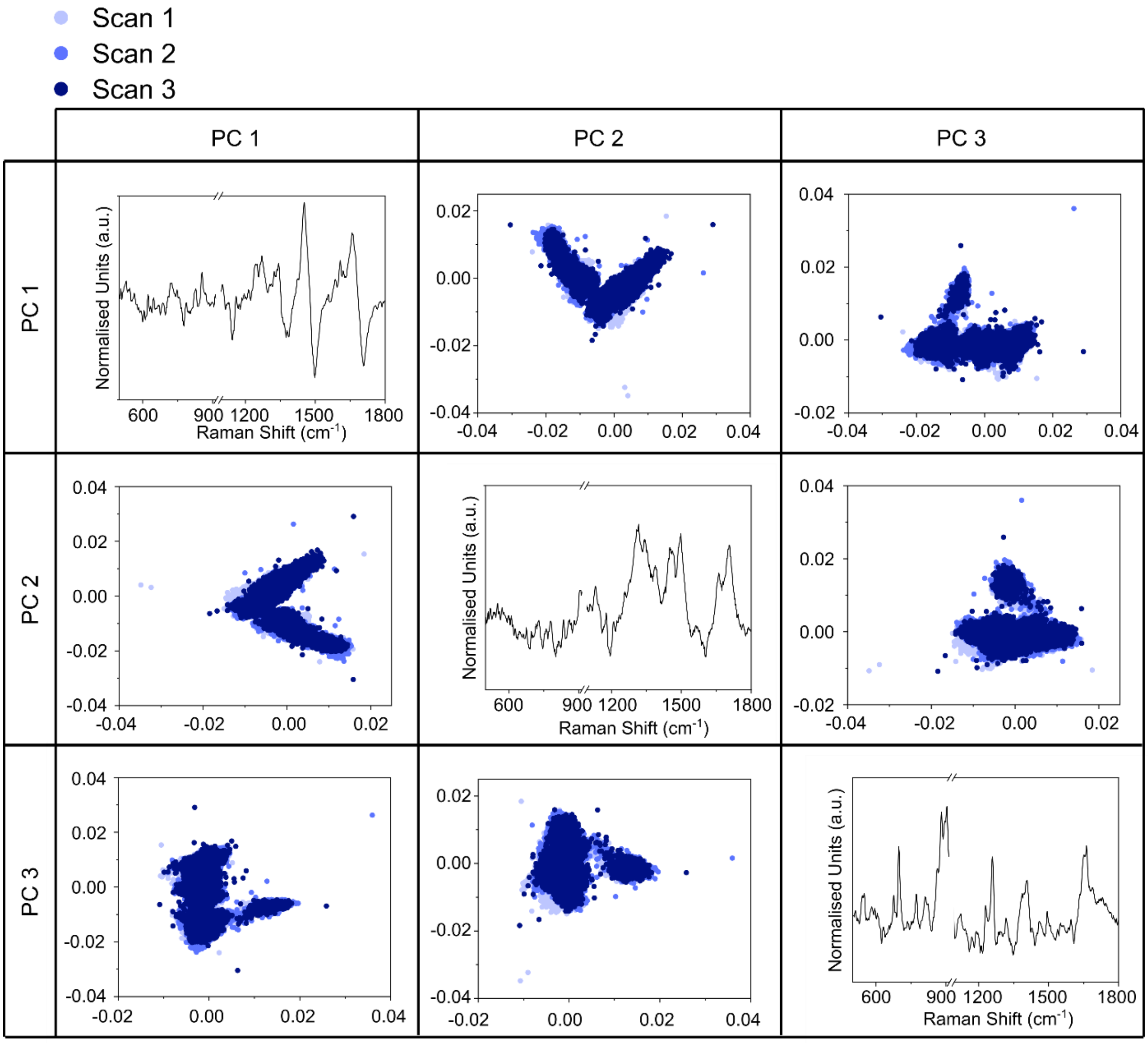

Supplementary Figure 5: Comparison of the Raman spectra generated from the tolerance study. Principal component analysis (PCA) performed on all spectra collected from the live tolerance scans of four independent zebrafish embryos (N = 4). The three main principal components (PC 1, PC 2, PC 3) are displayed. Data is shown for zebrafish embryos after the first scan (light blue markers), after the second scan (blue markers), and after the third scan (dark blue markers).

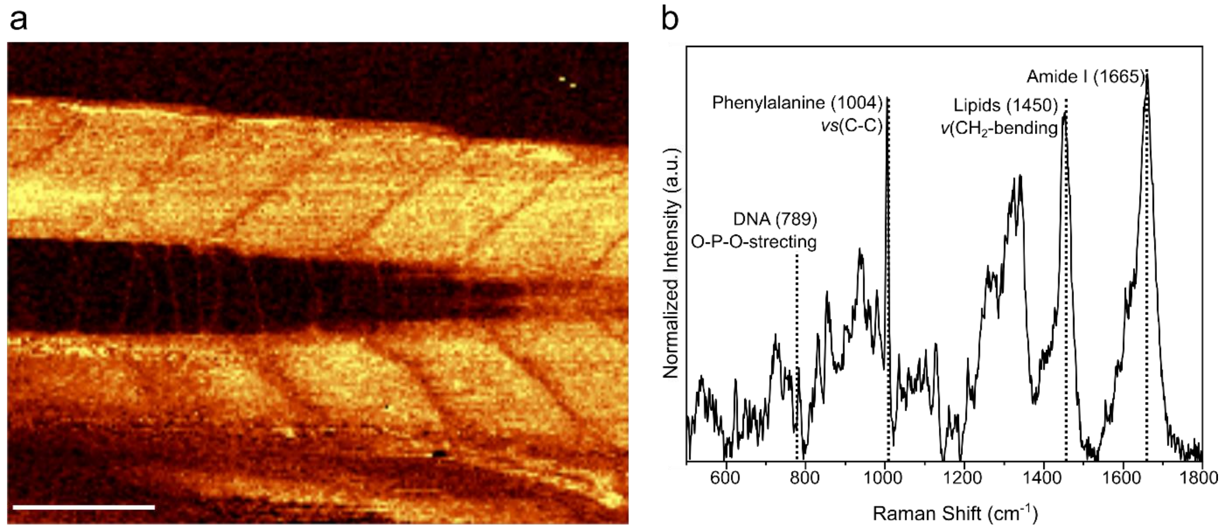

Supplementary Figure 6: Confocal Raman spectroscopic imaging of a living zebrafish embryo using an infrared laser source. a) Large area scan of a live zebrafish embryo using confocal Raman spectroscopic imaging (cRSI) with a 785 nm laser (N = 1). The image is generated by univariate analysis using the area under the peak at 1665 ± 20 cm^{-1}. Scale bar: 100 µm. **b)** Representative spectra obtained from the cRSI scan of the living zebrafish embryo muscle tissue, with dashed line highlighting peaks corresponding to key biomolecular features

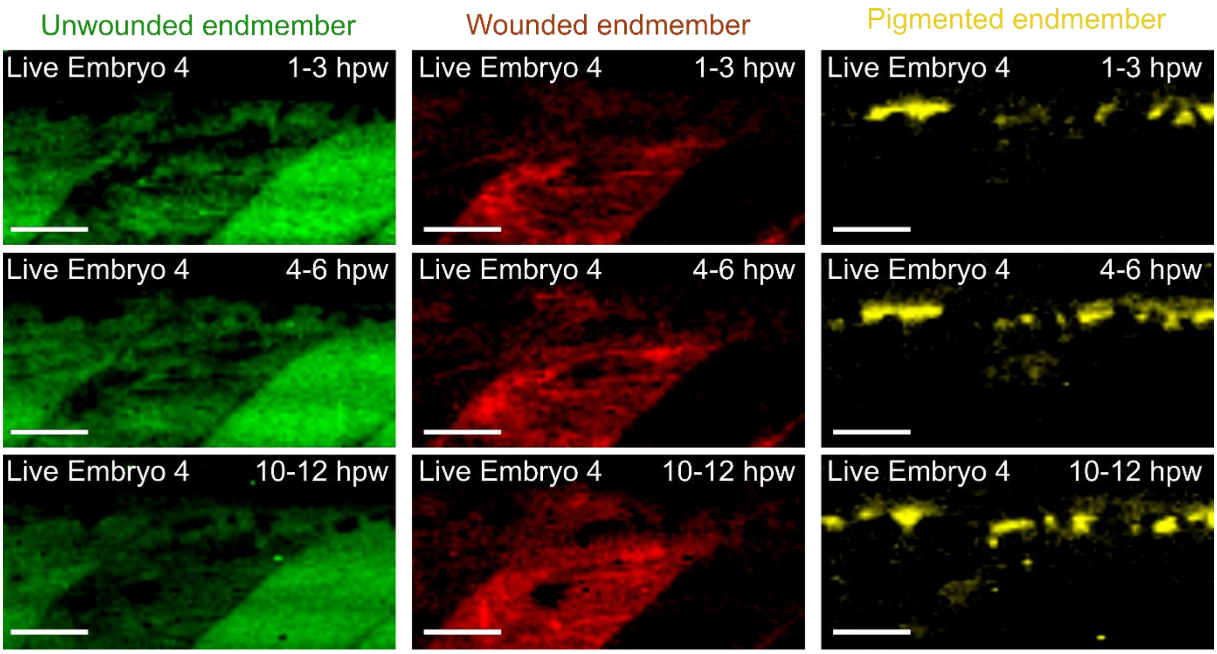

Supplementary Figure 7: **Time lapse confocal Raman spectroscopic imaging of a live zebrafish embryo after a stab wound in the epaxial myotome**. Images showing the distribution of the three tissue components identified by performing vertex component analysis (VCA) on confocal Raman spectroscopic imaging scans of a wounded zebrafish embryo (N = 1), one of a set of three live scanned embryos. Images were collected at three different intervals: 1-3, 4-6, 10-12 hours post wounding (hpw). Four identified endmembers are displayed: unwounded (green), wounded (red), pigment (yellow), and water (black). Scale bars: 40 µm.

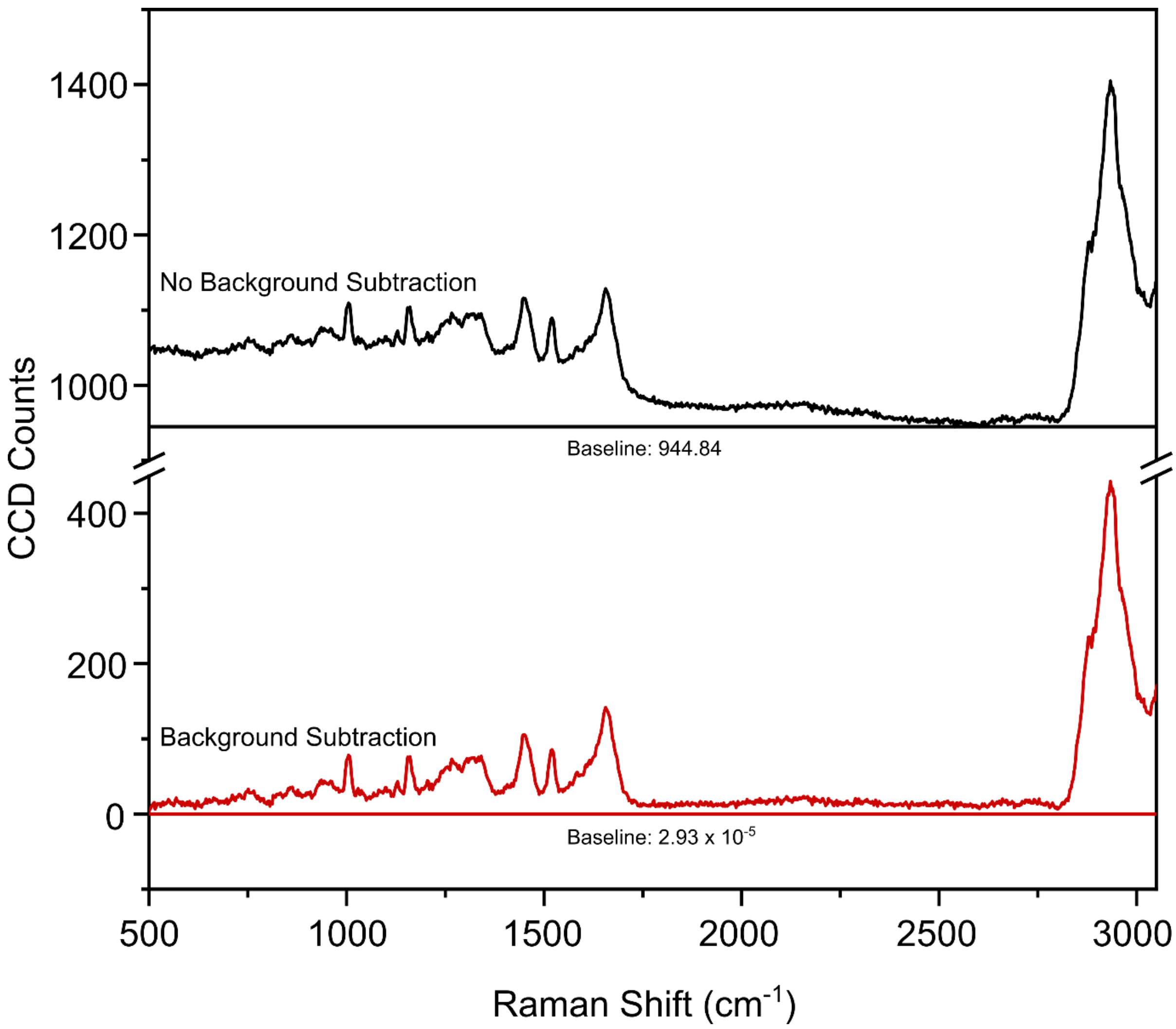

Supplementary Figure 8: Example of background subtraction performed on Raman spectra prior to data analysis. The black line shows an exemplar spectrum prior to background subtraction and the red line show the same spectrum after background subtraction. Each spectrum is shown with its calculated baseline to indicate their respective minima.

Supplementary Table 1: Annotated Peak Library of Raman Signals Detected in Zebrafish Embryos.

Biomolecule	Anatomical Region	Peak Center (cm^{-1})	Molecular Vibration	Reference
DNA	Whole Embryo	789	O-P-O stretching	37
Collagen	Muscle segments	852	Hydroxyproline	32
Collagen	Muscle segments	918	Hydroxyproline	32
Collagen	Muscle segments	935	C-C stretching	28
Proteins	Whole Embryo	1004	Ring breathing phenylalanine v_s(C-C)	32
Proteins Carbohydrates	Gut	1128	v(C-C)	32
Carotenoids	Surface/Blood	1159	C-C=C-C Conjugation	36
Proteins	Gut	1170	Tyrosine	35,32
Amide III (Collagen)	Gut	1246	v_s(C-N)	32
Lipids Collagen		1305	CH_2Twisting δ(CH_2)	29, 32
Collagen	Gut	1312	CH_3CH_2 twisting modes of collagen	30, 31
Phospholipids	Gut	1366	v_s(CH_3)	35
Lipids	Whole Embryo	1450	(CH_2 bending)	29
Carotenoids	Surface/Blood	1528	C-C=C-C Conjugation	27, 36
Cytochrome	Gut	1579	v(C=C)	38
Collagen	Gut	1587	Hydroxyproline	32
Collagen	Gut	1636	Amide I Band	35
Amide I	Whole Embryo	1665	Amide I	32
Lipids and Fatty Acids	Whole Embryo	1748	v(C=O)	29, 38
Lipid	Whole Embryo	2850/2	v_s(CH_2)	29,38,
Lipid	Whole Embryo	2885	v_s(CH_3)	29, 38,
Protein	Whole Embryo	2940	v_{as}(CH_2)	38
