# Supplementary material for: In vivo biomolecular imaging of zebrafish embryos using confocal Raman spectroscopy
Source: Nat Commun. 2020 Dec 2;11:6172. doi: 10.1038/s41467-020-19827-1 (PMC7710741; doi:10.1038/s41467-020-19827-1)
Supplement: Supplementary file 2 — Description of Additional Supplementary Files [file 41467_2020_19827_MOESM2_ESM.pdf]

Description of Additional Supplementary Files

File Name: Supplementary Movie 1
Description: Screenshot brightfield video of zebrafish embryo at 3 days post fertilization prior to live confocal Raman spectroscopic imaging (cRSI) using a 785 nm laser. N=4 embryos were used for qualitative assessment of changes in the blood circulation during live cRSI. No apparent changes were observed.

File Name: Supplementary Movie 2
Description: Screenshot brightfield video of zebrafish embryo at 3 days post fertilization after three consecutive live confocal Raman spectroscopic imaging (cRSI) scans using a 785 nm laser. N=4 embryos were used for qualitative assessment of changes in the blood circulation during live cRSI. No apparent changes were observed.
