# Supplementary material for: In vivo biomolecular imaging of zebrafish embryos using confocal Raman spectroscopy
Source: Nat Commun. 2020 Dec 2;11:6172. doi: 10.1038/s41467-020-19827-1 (PMC7710741; doi:10.1038/s41467-020-19827-1)
Supplement: Supplementary file 5 — Reporting Summary [file 41467_2020_19827_MOESM5_ESM.pdf]

nature research

Corresponding author(s): Molly M Stevens

Last updated by author(s): Oct 22, 2020

Reporting Summary

Nature Research wishes to improve the reproducibility of the work that we publish. This form provides structure for consistency and transparency in reporting. For further information on Nature Research policies, see our Editorial Policies and the Editorial Policy Checklist.

Statistics

For all statistical analyses, confirm that the following items are present in the figure legend, table legend, main text, or Methods section.

n/a	Confirmed	
☐	☒	The exact sample size (*n*) for each experimental group/condition, given as a discrete number and unit of measurement
☐	☒	A statement on whether measurements were taken from distinct samples or whether the same sample was measured repeatedly
☐	☒	The statistical test(s) used AND whether they are one- or two-sided<br>*Only common tests should be described solely by name; describe more complex techniques in the Methods section.*
☐	☒	A description of all covariates tested
☐	☒	A description of any assumptions or corrections, such as tests of normality and adjustment for multiple comparisons
☐	☒	A full description of the statistical parameters including central tendency (e.g. means) or other basic estimates (e.g. regression coefficient) AND variation (e.g. standard deviation) or associated estimates of uncertainty (e.g. confidence intervals)
☐	☒	For null hypothesis testing, the test statistic (e.g. *F*, *t*, *r*) with confidence intervals, effect sizes, degrees of freedom and *P* value noted<br>*Give P values as exact values whenever suitable.*
☒	☐	For Bayesian analysis, information on the choice of priors and Markov chain Monte Carlo settings
☒	☐	For hierarchical and complex designs, identification of the appropriate level for tests and full reporting of outcomes
☒	☐	Estimates of effect sizes (e.g. Cohen's *d*, Pearson's *r*), indicating how they were calculated

Our web collection on statistics for biologists contains articles on many of the points above.

Software and code

Policy information about availability of computer code

Data collection	Control Four (WITec) was used for collection of Raman spectroscopic data, TEM images were collected using DigitalMicrograph (Gatan) and FEI Software (version1.6.4), Fluorescent confocal microscopy images were collected using Leica Application Suite Advanced Fluorescence 2.7.3.9723
Data analysis	Project Four software (WITec) was used for processing of Raman Spectroscopic data. ICY (Version 2.0.3.0) and ImageJ (ImageJ 2.0.0-rc-69/1.52i / Java 1.8.0_172 (64-bit) (NIH)) was used for all other image analysis. Python and MATLAB were used for stastical analysis. Microsoft Excel (Version 16) and Origin (Version 2018b) were used for all data analysis and statistics. Screen recorder (Icecream Apps Version: 5.0). MATLAB codes used for analysis in this paper is available at: https://github.com/conor-horgan/Raman-Zebrafish-Analysis

For manuscripts utilizing custom algorithms or software that are central to the research but not yet described in published literature, software must be made available to editors and reviewers. We strongly encourage code deposition in a community repository (e.g. GitHub). See the Nature Research guidelines for submitting code & software for further information.

Data

Policy information about availability of data

All manuscripts must include a data availability statement. This statement should provide the following information, where applicable:

- Accession codes, unique identifiers, or web links for publicly available datasets
- A list of figures that have associated raw data
- A description of any restrictions on data availability

Raw data are available online at DOI: 10.5281/zenodo.4059924.

Field-specific reporting

Please select the one below that is the best fit for your research. If you are not sure, read the appropriate sections before making your selection.

☒ Life sciences ☐ Behavioural & social sciences ☐ Ecological, evolutionary & environmental sciences

For a reference copy of the document with all sections, see nature.com/documents/nr-reporting-summary-flat.pdf

Life sciences study design

All studies must disclose on these points even when the disclosure is negative.

Sample size	Sample size is indicated in the figure legend for each experiment. No sample-size calculations were performed. Sample size were chosen based on previous experience with Raman spectroscopic analysis and determined to be adequate based on the magnitude and consistency of measurable differences between groups.
Data exclusions	No data were excluded from the analysis.
Replication	All experiments were repeated independently at least three times with similar results, apart from the full embryo 3D scan (N=1) and high-resolution imaging of the tail and gut (N = 1), TEM of mycobacterial infection (N=2) and the large area scan of living zebrafish (N=1). In addition, wound response experiment were successfully repeated on N=3 independent embryos, but the vertex component analysis was only performed on N=1 representative dataset.
Randomization	Samples were randomly allocated to each experimental group prior to any experiment involving more than one group.
Blinding	The investigators were not blinded to the groups and treatments during experiments. Blinding was not necessary because all data reported for these experiments based on the quantitative assays and are therefore not subjective.

Reporting for specific materials, systems and methods

We require information from authors about some types of materials, experimental systems and methods used in many studies. Here, indicate whether each material, system or method listed is relevant to your study. If you are not sure if a list item applies to your research, read the appropriate section before selecting a response.

Materials & experimental systems

n/a	Involved in the study	
☒	☐	Antibodies
☒	☐	Eukaryotic cell lines
☒	☐	Palaeontology and archaeology
☐	☒	Animals and other organisms
☒	☐	Human research participants
☒	☐	Clinical data
☒	☐	Dual use research of concern

Methods

n/a	Involved in the study	
☒	☐	ChIP-seq
☒	☐	Flow cytometry
☒	☐	MRI-based neuroimaging

Animals and other organisms

Policy information about studies involving animals; ARRIVE guidelines recommended for reporting animal research

Laboratory animals	Zebrafish (Danio rerio) embryos (TraNac mutant fish obtained from Julian Lewis, London Research Institute, London). Experiments involving zebrafish were conducted in accordance with UK Home Office requirements (Animals Scientific Procedures Act 1986, project licence P5D71E9B0). All experiments were conducted up to 5 days post fertilization, except for M. marinum experiments, where fish were kept until 6 days post fertilization according to (Project licenses: PPL P84A89400 and P4E664E3C). All experiments were conducted on embryos that were 3 days old apart from M. marinum infected embryos were the experiments were conducted at fish that were 6 days old. The gender of the embryos used in this study was not known.
Wild animals	The study did not involve wild animals.
Field-collected samples	The study did not involve field-collected samples.
Ethics oversight	Animal experiments were performed according to the Animals Scientific Procedures Act 1986 and approved by the Home Office (PPL 70/7700 & PPL 70/8365) & (Project licenses: PPL P84A89400 and P4E664E3C).

Note that full information on the approval of the study protocol must also be provided in the manuscript.
